# Characteristics and genetic diversity of multi-drug resistant extended-spectrum beta-lactamase (ESBL)-producing *Escherichia coli* isolated from bovine mastitis

**DOI:** 10.18632/oncotarget.21496

**Published:** 2017-10-04

**Authors:** Tariq Ali, Sadeeq ur Rahman, Limei Zhang, Muhammad Shahid, Dandan Han, Jian Gao, Shiyao Zhang, Pamela L. Ruegg, Umer Saddique, Bo Han

**Affiliations:** ^1^ Department of Clinical Veterinary Medicine, College of Veterinary Medicine, China Agricultural University, Beijing, P.R. China; ^2^ College of Veterinary Sciences and Animal Husbandry, Abdul Wali Khan University, Garden Campus, Mardan, Pakistan; ^3^ Department of Dairy Science, University of Wisconsin, Madison, WI, USA; ^4^ Department of Animal Health, The University of Agriculture, Peshawar, Pakistan

**Keywords:** *E. coli*, ESBL, multilocus sequence typing, PCR-based replicon typing, split network analysis

## Abstract

A characterization of the drug resistance profiles, identification of PCR-based replicon typing, and multilocus sequence typing (MLST) and analysis of 46 ESBL-producing *Escherichia coli* from cows with mastitis are described. All multidrug-resistant isolates of various phylogenetic groups (A = 31, B1= 3, B2 = 2, D = 10) were ESBL-producers of genotypes CTX-M-15 (29), CTX-M-55 (4), CTX-M-14 (4), CTX-M-3 (1), CTX-M-1 (1), TEM (22) and SHV (8) that were found on conjugative plasmids of diverse incompatibility groups (primarily IncF). Transconjugation experiments indicated successful (100%) trans-conjugation, which was verified phenotypically and genotypically. A total of 28 sequence types (ST) were identified, with 10% of isolates being ST410, and 9 other ST that were assigned arbitrary numbers, reflecting the degree of diversity. Multilocus sequence analysis revealed two lineages, a dominant and a small lineage. Split-decomposition showed intraspecies recombination clearly contributed in genetic recombination generating genotypic diversity among the isolates, and a lack of interspecies recombination. This coherent analysis on genetic structure of multidrug-resistant pathogenic *E. coli* population isolated from mastitic-milk weaponized with resistance elements from a large, rapidly developing country will be a helpful contribution for epidemiology and surveillance of drug resistance patterns, and understanding their global diversity.

## INTRODUCTION

*Escherichia coli* has been classified as a major pathogen causing bovine mastitis, inflammation of the mammary gland, due to its abundant and predominant isolation from infected tissues [1]. Other than mastitis, *E. coli* is also the most common pathogen responsible for several other serious diverse gastrointestinal or urinary tract infections, and even bacteremia in human beings, thereby causing millions of deaths every year around the globe. To eliminate this pathogen during infection, antimicrobial agents are administered for treatment, although in livestock these drugs may also be used in prophylactically manner, to prevent bacterial infections or promote livestock growth. To avoid mastitis-associated economic losses in dairy cows, potent drugs such as extended-spectrum cephalosporins are often preferred for treatment, this trend may be associated with emergence of bacterial resistance as indicated by the increasing prevalence of resistant extended-spectrum beta-lactamase (ESBL)-producing *Enterobacteriaceae*, especially *E. coli*, isolated from dairy and food-producing animals in China [2–4]. Notably, the emergence of novel antibacterial resistance mechanisms [5] and prevalence of multidrug resistant *E. coli* isolated from food animals have steadily increased over the last decades in China [6] and around the globe [7]. Although persistent and blind use of antimicrobials is likely the most important factor providing selective pressure for emergence of traits associated with resistance, understanding mechanisms of dissemination and genetic tools that help bacteria in spreading such elements are vital to control and elimination of antibiotic resistance.

Genes encoding ESBL have been classified into three major categories: *bla*_CTX-M_, *bla*_SHV_ and *bla*_TEM_. The *bla*_CTX-M_ type has been further distributed into five main sub-groups (*bla*_CTX-M-1_, *bla*_CTX-M-2_, *bla*_CTX-M-8_, *bla*_CTX-M-9_, *bla*_CTX-M-25_) and different variants (approximately more than 150 CTX-M variants) have been documented thus far (http://www.lahey.org/studies). Previous reports suggest that the *bla*_CTX-M_ type, predominantly *bla*_CTX-M-15_, was the most prevalent ESBL type around the world and especially in Asia [8]. Notably, *E. coli* producing *bla*_CTX-M-15_ ESBL is an important cause of health care oriented- as well as community based-infections in humans [9], and has also been increasingly reported recently from food producing animals [2]. It has been suggested that acquiring plasmids harbouring *bla*_CTX-M_ and other ESBL encoding genes is the predominant mechanism associated with the increase of ESBL-producing *E. coli*. Importantly, ESBL genes on plasmids have been more frequently reported linked with certain plasmid replicon typing, including IncF, IncI, IncN, IncHI2, IncK and IncL/M groups [10]. The IncF group (FIA, FIB and FII) including IncK and IncI1 largely contribute to the dissemination of ESBL genes, primarily of *bla*_CTX-M -15_, while the *bla*_CTX-M -3_ gene is carried by plasmids of IncL/M and IncI1, and IncHI2 plasmids harbour *bla*_CTX-M -9_ [10].

For the purpose of precise identification and typing of *E. coli*, numerous molecular typing approaches have been developed including pulsed-field gel electrophoresis, restriction fragment length analysis, amplified fragment length fingerprinting, whole cell protein analysis, matrix-assisted laser desorption ionization, time of flight mass spectrometry, and multilocus sequence typing (MLST) [11–13]. Although all these methods can identify *E. coli*, MLST gives high resolution at specie level as compared to other molecular methods, and is accepted worldwide and widely used for typing of *E. coli* [14]. The MLST method for *E. coli* formulated in 2006 and updated in 2007 is used worldwide for typing and assessing the population structure of *E. coli* [15]. Furthermore, phylogenetic algorithm and procedures implemented in the multilocus sequence analysis (MLSA) based on the nucleotide sequences of allelic locus used in the MLST are being reported widely used for identification and inferring phylogenetic relationships between isolates. Moreover, MLST has been proven a powerful tool for insights into the population structure and recombination analysis of many bacterial pathogens including *E. coli* [16, 17]. Nevertheless, MLST studies on drug resistant *E. coli*, especially ESBL-producing, isolated from food animals in general and particularly from mastitic dairy cows are lacking. We recently reported on the high prevalence of multi-drug resistant ESBL-producing *E. coli* from milk samples of dairy cows suffering from bovine mastitis [4]. In this study, we extended our analysis on the drug resistance profile including minimum inhibitory concentrations (MIC), plasmid replicon typing, and further provided insight based on the MLST of previously reported and newly isolated *E. coli* isolates. For the first time in China, *E. coli* isolates from bovine mastitic milk were identified as the species of *E. coli* by using MLSA, extrapolate about the recombination features and genetic diversity that reveals detailed insights into the prevailing drug resistant worldwide disseminated pathogen.

## RESULTS

### Prevalence and genotypes of ESBL-producing *E. coli*

As a part of our project, during 2015-2016 we screened 1440 milk samples from cows suffering from mastitis belonged to 69 different dairy herds in various provinces of China (Supplementary Table 1). Of these mastitic milk samples, a total of 181 *E. coli* isolates were recovered. Among these isolates, 46 were confirmed as extended-spectrum beta-lactamase (ESBL)-producers. Genotyping of these ESBL-producing *E. coli* by specific PCR assay showed that CTX-M producing genotypes were predominant (*n* = 39), followed by TEM (*n* = 22) and SHV (*n* = 8). Further sequencing of CTX-M genotypes revealed that CTX-M-15 was the most dominant allele, which was harbored by 29 isolates, followed by CTX-M-55 (*n* = 4), CTX-M-14 (*n* = 4), CTX-M-3 (*n* = 2) and CTX-M-1 (*n* = 1). All TEM and SHV alleles were found identical to TEM-1 and SHV-1 except one of the isolate was carrying singlet SHV-12 obtained from a dairy cow in Heilongjiang province (Table 1). Genotypes TEM and SHV were found in combination with CTX-M-15 in the majority of isolates.

**Table 1:** Molecular characteristics and MLST typing of extended-spectrum beta-lactamase-producing *E. coli* isolated from bovine mastitis

Clinical Isolates	Location	ESBL types	Phylo- groups	Plasmid replicon types	ST	STC	Strain deposited in MLST data base	Resistance profiles
1-G	Guangdong	CTX-M-15	A	IncFrep, IncHI2, IncP, IncY	ST744	non	HTS01G	A, AC, CX, CTX, CAZ, FEP, AZT, N, CIP, C, ST, TE
2-G	Guangdong	CTX-M-15, TEM-1	A	IncHI2, IncK/B, IncY	ST761	non	-	A, AC, CX, CTX, CAZ, FEP, AZT, N, C, ST, TE
3-Im	Inner Mongolia	TEM-1, SHV-1	D	IncHI1, IncK/B	ST156	STC156	-	A, AC, CTX, N, CIP, C, ST, TE
4-Im	Inner Mongolia	CTX-M-15	A	IncFIA, IncFrep	ST906	non	-	A, AC, CX, CTX, CAZ, AZT, N, CIP, C, ST, TE, G
5-Im	Inner Mongolia	CTX-M-15, TEM-1, SHV-1	D	IncFIA, IncFIB, IncK/B	ST2008	non	HTS05IM	A, AC, CX, FOX, CTX, CAZ, FEP, AZT, N, CIP, ST, TE, G
12-Im	Inner Mongolia	CTX-M-15, TEM-1	B1	IncFIB, IncFIIs, IncFrep, IncI1, IncK/B	ST2008	non	HTS12IM	A, AC, CX, FOX, CTX, CAZ, FEP, AZT, N, CIP, ST, TE, G
14-Im	Inner Mongolia	CTX-M-15, SHV-1	A	IncFrep, IncK/B	ST1121	non		A, AC, CX, CTX, CAZ, AZT, N, C, CIP, ST, TE, G
17-Im	Inner Mongolia	CTX-M-15	A	IncFIA, IncFIB, IncHI2, IncN	ST361	non	HTS17IM	A, AC, CX, CTX, CAZ, FEP, AZT, N, CIP, ST, TE, G
18-Im	Inner Mongolia	CTX-M-15	A	IncFIA, IncFIB, IncFrep, IncK/B	ST361	non	-	A, AC, CX, CTX, CAZ, AZT, N, CIP, ST, TE, G
19-Im	Inner Mongolia	CTX-M-15	A	IncFIA, IncFIB, IncHI2, IncK/B	ST1121	non	HTS19IM	A, AC, CX, CTX, CAZ, FEP, AZT, N, CIP, ST, TE, G
20-Im	Inner Mongolia	CTX-M-15	D	IncFIA	ST468	non	HTS20IM	
21-Im	Inner Mongolia	CTX-M-15, SHV-1	D	IncFrep	ST3476	non	HTS21IM	CTX, CIP, TE
22-Im	Inner Mongolia	CTX-M-15, SHV-1	D	IncFIB, IncFrep, IncK/B	ST410	STC23	HTS22IM	A, AC, CX, FOX, CTX, CAZ, FEP, AZT, N, CIP, ST, TE, G
23-Im	Inner Mongolia	CTX-M-15, SHV-1	A	IncFIA, IncFIB, IncFrep, IncN, IncK/B	ST1221	non	HTS23IM	A, AC, CX, CTX, CAZ, AZT, N, C, CIP, ST, TE, G
25-Im	Inner Mongolia	CTX-M-15, TEM-1, SHV-1	A	IncFIA, IncFIB, IncFrep, IncK/B	ST361	non	HTS25IM	A, AC, CX, FOX, CTX, FEP, AZT, N, CIP, ST, TE, G
28-Im	Inner Mongolia	CTX-M-15, TEM-1	A	IncFIB, IncFrep, IncP	ST3044	non	-	A, AC, CX, FOX, CTX, CAZ, AZT, N, CIP, ST, TE, G
32-Im	Inner Mongolia	CTX-M-15	A	IncFIB, IncFrep, IncHI2, IncN	ST2008	non	-	A, AC, CX, CTX, CAZ, FEP, C
1-Im2	Inner Mongolia	CTX-M-14	A	IncI1, IncY	ST2521	non	HTS01IM2	A, CX, CTX, AZT,
2-Im2	Inner Mongolia	TEM-1	A	IncHI1, IncFIB	ST4085	non	HTS02IM2	A, CX, CTX, G
3-Im2	Inner Mongolia	TEM-1	A	IncFIA, IncFIB, IncFrep	ST392	non	HTS03IM2	CX, CTX, AZT
4-Im2	Inner Mongolia	CTX-M-15	A	IncFIB, IncFrep, IncHI2	ST1080	non	HTS04IM2	A, CX, CTX, AZT, C, ST, G
5-Im2	Inner Mongolia	CTX-M-15	A	IncFIB, IncFrep	ST2035	non	HTS05IM2	A, CX, CTX, AZT, N
7-Im2	Inner Mongolia	CTX-M-55, TEM-1	B1	IncFIB, IncFrep, IncN	ST410	STC23	HTS07IM2	A, AC, CX, CTX, CAZ, FEP, AZT, N, C, CIP, ST, TE, G
8-Im2	Inner Mongolia	CTX-M-14	A	IncFIB, IncFrep	ST410	STC23	-	A, CX, CTX, CAZ, FEP, AZT, N, C, CIP, ST, TE, G
9-Im2	Inner Mongolia	CTX-M-55, TEM-1	A	IncFIB, IncFrep, IncN	ST410	STC23	-	A, CX, CTX, CAZ, FEP, AZT, N, C, ST, TE, G
10-Im2	Inner Mongolia	CTX-M-15, TEM-1	A	IncFIA, IncFIB, IncFrep, IncH2	ST4085	non	-	A, CX, CTX, CAZ, AZT, N, C, CIP, ST, TE, G
11-Im2	Inner Mongolia	TEM-1	B1	IncFIB, IncFrep, IncH2	ST4085	non	HTS11IM2	A, AC, CX, CTX, AZT, N, C, ST, TE, G
12-Im2	Inner Mongolia	CTX-M-55	A	IncFIB, IncFrep, IncH2	ST215	STC10	-	A, CX, CTX, FEP, AZT, N, C, CIP, TE
8-Im3	Inner Mongolia	CTX-M-55	D	IncFIB, IncFIC, IncFrep	ST5746	non	HTS08IM3	A, AC, CX, CTX, AZT, N, C, ST, TE, G
1-Im5	Inner Mongolia	CTX-M-15, TEM-1	A	IncFrep	ST744	non	HTS01IM5	A, AC, CX, CTX, CAZ, FEP, AZT, N, C, CIP, ST, TE, G
2-Im5	Inner Mongolia	TEM-1	A	IncFIB, IncFrep, IncH2	ST4085	non	HTS02IM5	A, AC, CX, FOX, CTX, CAZ, FEP, AZT, N, C, CIP, ST, TE, G
3-Im5	Inner Mongolia	CTX-M-14, TEM-1	A	IncFIA, IncFIB, IncFrep, IncP	ST58	STC155	HTS03IM5	A, AC, CX, FOX, CTX, N, ST, TE, G
1-Im6	Inner Mongolia	CTX-M-15	B2	IncFIB, IncFrep	ST117	non	HTS01IM6	A, AC, CX, CTX, CAZ, FEP, AZT, N, C, ST, TE, G
2-Im6	Inner Mongolia	CTX-M-15	B2	IncFIB, IncFrep	ST117	non	-	A, AC, CX, CTX, CAZ, FEP, AZT, N, C, CIP, ST, TE, G
4-N	Jiangsu	CTX-M-15, TEM-1	D	IncI1, IncY	ST88	STC23	HTS04N	A, CX, CTX, CAZ, FEP, AZT, N, C, ST, TE, G
5-N	Jiangsu	CTX-M-15, TEM-1	D	IncI1, IncY	ST88	STC23	-	A, CX, CTX, CAZ, FEP, AZT, N, C, ST, TE, G
4-Hb_2_	Hebei	TEM-1	D	IncFIA, IncY, IncL/M	ST69	STC69	-	A, AC, CX, CTX, CAZ, FEP, AZT, N, C, ST, TE, G
1-Hb_3_	Hebei	CTX-M-15	A	IncFIB, IncFrep	ST58	STC155	HTS01HB	A, AC, CX, CTX, CAZ, FEP, AZT, N, C, CIP, ST, TE, G
1-L	Liaoning	CTX-M-15, TEM-1	A	IncFIB, IncN	ST117	non	HTS01L	A, AC, CX, CTX, CAZ, FEP, AZT, N, C, ST, TE, G
8-L2	Liaoning	CTX-M-14	A	IncFIB, IncHI2	ST3951	non	-	A, AC, CX, CTX, CAZ, AZT, N, ST, TE
N-1	Ningxia	CTX-M-3, TEM-1	A	IncFIA, IncI1	NT			A, AC, CX, CTX, N, C, TE
H-5	Heilongjiang	SHV-12	A	IncFIB,	NT			A, CX, FOX, CTX
Hn-6	Henan	CTX-M-15	A	IncFrep, IncK/B	NT			A, AC, CX, FOX, CTX, C, TE
2-Hn1	Henan	CTX-M-1	A	IncFIB, IncFIC, IncK/B	ST58	STC155	-	A, CX, CTX, CAZ, FEP, AZT, N, C, G
6-Hn1	Henan	CTX-M-15, TEM-1	A	IncFIB, IncK/B	ST6482	non	-	A, AC, CX, CTX, CAZ, FEP, AZT, N, ST, TE, G
7-Hn1	Henan	CTX-M-15, TEM-1	A	IncFIA, IncFIB, IncFrep, IncK/B	ST540	non	HTS07HN	A, AC, CX, FOX, CTX, CAZ, FEP, AZT, N, ST, TE, G

NT: nontypeable; A: ampicillin, AC: amoxicillin/clavulanic acid, CX: cephalexin, FOX: cefoxatin, CTX: cefotaxime, CAZ: ceftazidime, FEP: cefepime, AZT: aztreonam, N: nalidixic acid, ST: trimethoprim/sulphamethoxazole, TE: tetracycline, GM: gentamicin.

### Phylogenetic grouping

Using triplex PCR assay, the 46 ESBL-producing *E. coli* were distributed to the phylogenetic groups A (*n* =31), B1 (*n*= 3), B2 (*n* = 2) and D (*n* =10) as shown in Table 1.

### Antimicrobial resistance in ESBL-producers

**Figure 1:**
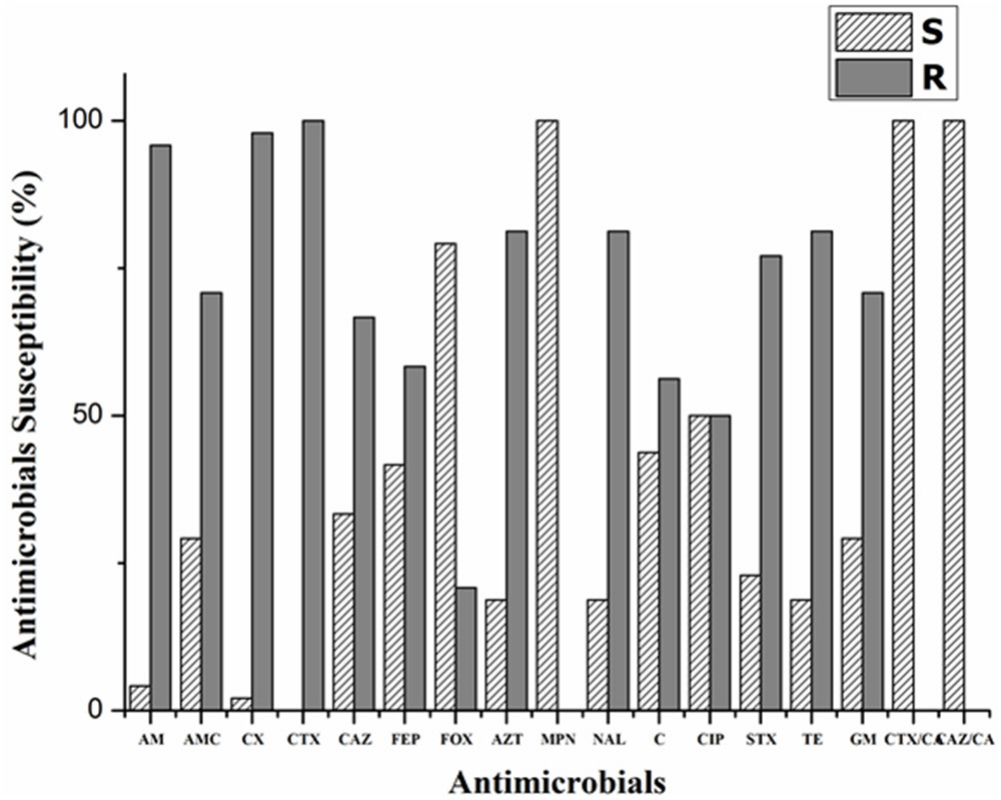
Antibiotic susceptibility profiles and PCR-based plasmid replicon typing of extended-spectrum beta-lactamase-producing *E. coli* isolates from bovine mastitis Results of antimicrobials susceptibility testing of ESBL-producing representative *E. coli* isolates are shown. The isolate(s) was tested for ESBL-production as described by CLSI. AM: ampicillin, AMC: amoxicillin/clavulanic acid, CX: cephalexin, cefaclor, CTX: cefotaxime, CAZ: ceftazidime, FEP: cefepime, FOX: cefoxatin, AZT: aztreonam, MPN: meropenem, NAL: nalidixic acid, C: chloramphenicol, CIP: ciprofloxacin, STX: trimethoprim/sulphamethoxazole, TE: tetracycline, GM: gentamicin, CTX/CA: cefotaxime/ clavulanic acid and CAZ/CA: ceftazidime/clavulanic acid.

Dairy cattle with mastitis are treated with broad spectrum antibiotics, so we screened these isolates for resistance against commonly used antibiotics. Interestingly, all the 46 ESBL-producing *E. coli* were multidrug resistant phenotypes (i.e., showing resistance to three or more classes of antimicrobial agents) by standard disc diffusion (Figure 1) and MIC (Table 2). On observing the antibiotic susceptibility patterns of ESBL-producers, all isolates were non-susceptible to cefotaxime, while 32 (66.67%) and 28 (58.33%) isolates were resistant to ceftazidime and cefepime, respectively. Resistance to other classes of antimicrobial agents was also greater (Figure 1). However, all isolates were susceptible to meropenem and 38 (79.17%) isolates were susceptible to cefoxatin (MIC of various drugs tested are shown in Table 2), confirming the level of resistance determined initially by double disc synergy test. Isolates under study were resistant revealing highest values of MIC 50 and MIC 90 of common drugs in clinical practice such as ampicillin, cefotaxime, ceftriaxone, tetracycline, gentamycin *etc*. (Table 2).

**Table 2:** Minimum inhibitory concatenations (MIC) of ESBL-producing *E. coli* (*n* = 46)

Antimicrobial agents	Break-points^a^ (ug/ml)	MIC range (ug/ml)	MIC_50_ (ug/ml)	MIC_90_ (ug/ml)	No. of susceptible isolates (%)	No. of resistant isolates (%)
Ampicillin	≥ 32	0.5 - > 256	>256	>256	1 (2.17)	45 (97.83)
Cefazolin	≥ 8	0.5 - > 256	>256	>256	2 (4.35)	44 (95.65)
Cefoxatin	≥ 4	0.5 - > 256	>1	>64	31 (67.39)	15 (32.619)
Cefotaxime	≥ 4	0.5 - > 256	>256	>256	0 (00)	46 (100)
Ceftriaxone	≥ 4	0.5 - > 256	>256	>256	2 (4.35)	44 (95.65)
Ciprofloxacin	≥ 4	0.5 - > 256	>2	>256	22 (47.83)	24 (52.17)
Norfloxacin	≥ 16	0.5 - > 256	>32	>256	20 (43.48)	26 (56.52)
Amikacin	≥ 64	0.5 - > 256	>64	>128	21 (45.65)	25 (54.35)
Gentamicin	≥ 16	0.5 - > 256	>128	>256	10 (21.74)	36 (78.26)
Kanamycin	≥ 64	0.5 - > 256	>128	>256	10 (21.74)	36 (78.26)
Tetracycline	≥ 16	0.5 - > 256	>256	>256	6 (13.04)	40 (86.96)
Chloramphenicol	≥ 32	0.5 - > 256	>128	>256	15 (32.61)	31 (67.39)
Trimethoprim	≥ 16	0.5 - > 256	>256	>256	8 (17.39)	38 (82.61)

^a^CLSI, 2015.

### PCR-based plasmid replicon typing

**Figure 2:**
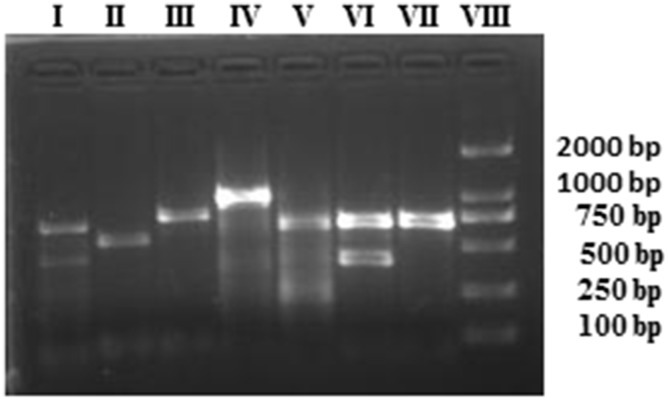
Detection of plasmid replicon types in ESBL-producing *E. coli* using PCR assay The amplified products were run on 2% agarose gel. Lane I= IncFIB and IncIncFIA, Lane II = IncN, Lane III =IncY, Lane IV = Inc L/M, Lane V = IncHI2, Lane VI = IncFIB and IncHI1, Lane VII = IncFIB, and Lane VIII = molecular marker (2000 bp).

Identification of the plasmid replicon typing for epidemiological studies is quite important. The results of PCR-based replicon typing of all the ESBL-producing *E. coli* revealed that IncF was the major plasmid replicon type identified in 41 isolates, whereas IncL/M was detected in only one isolate. Figure 2 shows the results of plasmid replicon types of some of the ESBL-producing *E. coli* isolates from bovine mastitis. The plasmids belong to the incompatibility groups IncFIA (*n* = 14), IncFIB (*n* = 31), IncFIC (*n* = 2), IncFrep (*n* = 29), IncFIIs (*n* = 1), IncH1 (*n* = 2), IncH2 (*n* = 11), IncI1 (*n* = 5), IncK/B (*n* = 12), IncN (*n* = 6), IncL/M (*n* = 1) and IncY (*n* = 8) as shown in the Table 1. Interestingly, in the majority of isolates multiple plasmids replicon types were found except in three ESBL-producing isolates, which carried a single plasmid replicon type.

### Resistance transfer experiment

Presence of the drug resistance elements such as *bla*_CTX-M_ on conjugative plasmids is expected to facilitate fast dissemination of these elements. Thus, isolates under study were further tested by mating/conjugation experiments to verify the transferability and possible horizontal spread of ESBL genes and plasmids. For this purpose, we randomly tested twelve isolates. All resulting transconjugates displayed features of multidrug resistant phenotypes as of donor (Table 3). Further, the transconjugates also displayed greater MIC of cefotaxime and ceftazidime. ESBL encoding genes were PCR-amplified from the transconjugates suggesting presence of ESBL genes on conjugative plasmids. Plasmid replicon typing using PCR was also carried out on all transconjugates, which showed that plasmid incompatibility type incF was found in all transconjugates except the two (Trans-1-Im2 and Trans-4N) that carried incY (Table 3).

**Table 3:** Characteristics of transconjugates using *E. coli* J53 strain of (*n* = 12)

Transconjugates	ESBL phenotype by DDST	ESBL genotypes	Plasmid replicons	CTX MIC (mg/l)	CAZ MIC (mg/l)	Resistance phenotype
Trans-1G	ESBL	CTX-M-15, TEM-1	IncFrep	> 256	> 64	A, AC, CX, CTX, CAZ, FEP, AZT, N, CIP, TE
Trans-5-Im	ESBL	CTX-M-15	IncFIA, IncFIB	> 256	> 128	A, AC, CX, FOX, CTX, CAZ, FEP, AZT, ST
Trans-12-Im	ESBL	CTX-M-15	IncFIB	> 256	> 64	A, CX, FOX, CTX, CAZ, FEP, AZT, CIP, ST, GM
Trans-17-Im	ESBL	CTX-M-15	IncFIA, IncFIB	> 256	> 128	A, CX, CTX, CAZ, FEP, AZT, CIP, TE, GM
Trans-18-Im	ESBL	CTX-M-15	IncFIB	> 256	> 32	A, AC, CX, CTX, CAZ, AZT, N, CIP, ST, TE, GM
Trans-19-Im	ESBL	CTX-M-15	IncFIA, IncFIB	> 256	> 128	A, CX, CTX, CAZ, FEP, AZT, G
Trans-25-Im	ESBL	CTX-M-15, TEM-1	IncFIA, IncFIB	> 256	> 0.5	A, CX, FOX, CTX, FEP, AZT, N, CIP, ST, TE, GM
Trans-28-Im	ESBL	CTX-M-15	IncFrep	> 256	> 256	A, AC, CX, FOX, CTX, CAZ, AZT, N, CIP, ST, TE, GM
Trans-1-Im2	ESBL	CTX-M-14	IncY	> 16	> 1	A, CX, CTX, AZT
Trans-4-N	ESBL	CTX-M-15, TEM-1	IncY	> 256	> 64	A, CX, CTX, CAZ, FEP, AZT, ST, TE, GM
Trans-6-Hn	ESBL	CTX-M-15	IncFrep	> 64	> 1	A, CX, FOX, CTX, C, TE
Trans-8-L2	ESBL	CTX-M-14	IncFIB	> 256	> 64	A, CX, CTX, CAZ, AZT, TE

A: ampicillin, AC: amoxicillin/clavulanic acid, CX: cephalexin, FOX: cefoxatin, CTX: cefotaxime, CAZ: ceftazidime, AZT: aztreonam, N: nalidixic acid, ST: trimethoprim/sulphamethoxazole, TE: tetracycline, GM: gentamicin.

### Multilocus typing and population structure

A total of 28 sequence types (ST), including 9 possibly novel ST, were identified among 43 typeable *E. coli* multidrug resistant isolates recovered from mastitic milk. Three of the *E. coli* isolates could not be assigned with a ST as we were not able to amplify at least one of the house keeping genes. Location from where these isolates were obtained, genes conferring resistance to target drugs, plasmid replicon, ST and antibiogram are mentioned in Table 1. Furthermore, ST identified along with allelic profile of 43 typeable *E. coli* isolates are given in Table 4.

**Table 4:** *E. coli* isolates collection used in this study, their allele profiles and sequence type identified

ID	ST	STC	*adk*	*fumc*	*gyrB*	*icd*	*mdh*	*purA*	*recA*
1-G	744	Non	10	11	135	8	8	8	2
2-G	761	Non	10	11	5	8	8	8	2
3-Im	10000	STC156	575	29	32	16	11	8	44
4-Im	10001	non	577	4	3	16	11	8	6
5-Im	2008	non	6	6	5	136	11	8	6
12-Im	2008	non	6	6	5	136	11	8	6
14-Im	10002	non	6	4	503	159	9	23	7
17-Im	361	non	10	99	5	91	8	7	2
18-Im	361	non	10	99	5	91	8	7	2
19-Im	1121	non	6	4	4	159	9	23	7
20-Im	5442	non	468	11	4	8	8	8	2
21-Im	3476	non	10	11	4	8	291	8	2
22-Im	410	STC23	6	4	12	1	20	18	7
23-Im	1121	non	6	4	4	159	9	23	7
25-Im	361	non	10	99	5	91	8	7	2
28-Im	10003	non	10	820	613	91	8	7	2
32-Im	10004	non	6	6	5	136	11	8	2
1-Im2	2521	non	6	19	3	135	11	8	6
2-Im2	4085	non	10	7	5	8	8	35	2
3-Im2	392	non	6	6	14	18	7	7	71
4-Im2	1080	non	6	4	7	9	7	7	56
5-Im2	2035	non	6	8	4	225	9	23	7
7-Im2	410	STC23	6	4	12	1	20	18	7
8-Im2	410	STC23	6	4	12	1	20	18	7
9-Im2	410	STC23	6	4	12	1	20	18	7
10-Im2	4085	non	10	7	5	8	8	35	2
11-Im2	4085	non	10	7	5	8	8	35	2
12-Im2	215	STC10	10	11	4	8	8	18	2
8-Im3	5746	non	207	744	176	141	170	2	2
1-Im5	744	non	10	11	135	8	8	8	2
2-Im5	4085	non	10	7	5	8	8	35	2
3-Im5	58	STC155	6	4	4	16	24	8	14
I-Im6	117	non	20	45	41	43	5	32	2
2-Im6	117	non	20	45	41	43	5	32	2
4-N	10005	STC-23	6	4	12	1	20	12	7
5-N	10006	STC-23	575	4	12	1	20	12	7
4-Hb2	69	STC-69	21	35	27	6	5	5	4
1-Hb3	58	STC155	6	4	4	16	24	8	14
1-L	117	non	20	45	41	43	5	32	2
8-L2	10007	non	45	45	36	507	8	294	94
2-Hn1	58	STC155	6	4	4	16	24	8	14
6-Hn1	10008	non	6	843	57	1	8	8	2
7-Hn1	540	non	6	7	57	1	8	8	2

ST numbers from 10000-08 are arbitrary number for the purpose of analysis.

**Figure 3:**
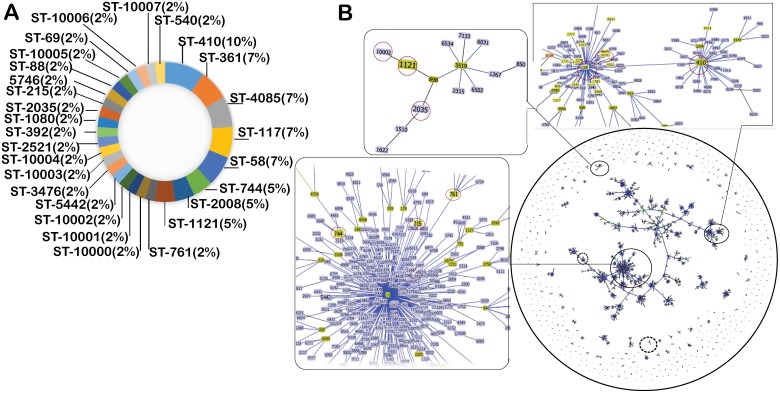
Sequence type diversity and population structure of ESBL-producing isolates **(A)** Pie chart representing sequence type diversity and their abundance among the 43 ESBL-positive *E. coli* isolates under study. **(B)** Population structure of resistant *E. coli* isolates. The goeBURST analysis of ~7000 ST available in the PubMLST database against the Achtman scheme. Each dot represents a single ST. Groups of the isolates were formed by linking the ST that are double locus variants (DLV) and named as clonal complex (CC). Snap shots of the three representative CCs zoomed in to indicate our under isolates (encircled). Representative larger size CCs that contain more than three types of our ST are shown with closed circle, while medium size CCs that contain two-to three of ST of our isolates are shown with dashed circle. For clarity reason, groups of CCs that contain a single type of our ST have not been demarcated. Snap shots of the three larger CCs, which are highlighted as closed circle, are zoomed to indicate CCs of our ST that are shown by red-circle. Light green-group founder; dark green-sub-group founder; light blue-common node.

The overall epidemiological information of the prevailing ST based on the MLST data of all isolates (*n* = 43) revealed a total of 28 ST, of which, 9 ST were assigned with arbitrary types (10000-08) and 19 ST were matched with the MLST database of *E. coli.* Sequence types 410 (10%), 361 (7%), 4085 (7%), 117 (7%), 58 (7%), 744 (5%), 2008 (5%) and 1121 (5%) were the most prevalent, while ST 761, 5442, 3476, 2521, 392, 1080, 2035, 215, 5746, 88 and 69 were the least (2%) prevalent. Interestingly, all those ST (10000-08) that could not be assigned as a result of at least a singular novel allele were all found among the least prevalent (2%) ST. Details about the distribution and abundance of ST are described in Figure 3A. The most prevalent ST (ST410) originated from Inner Mongolia, and 6 of the 9 ST that were not assigned based on the allelic profile also originated from Inner Mongolia. Furthermore, both of the isolates (ST10005 and ST10006) that could not be assigned to a ST originated from Jiangsu province.

All 28 ST found in the current report were grouped into 5 BURST group (BG) and 17 singletons types by eBURST analysis (Supplementary Table 3). The major BG contained 6 isolates and 3 ST (88, 410 and 10005) with a predicted founder of ST88. A second BG consisted of 3 isolates and 2 ST (744 and 761) with no predicted founder. BG 3 was comprised of 3 isolates and 2 ST (10007 and 540) with no predicted founder, BG4 was comprised of 3 isolates and 2 ST (2008, 10004) and BG 5 also comprised of 3 isolates and 2 ST (1121 and 10002), respectively (Supplementary Table 3). The geoBURST analysis with all 7256 ST available in the PubMLST database (as of March 15, 2017) including ST under this report (Figure 3B) revealed that ST2035 was linked to ST10002, ST2035, ST898 (Founder), ST1121 (founder) and ST3519 (founder). Interestingly, ST88 (encircled in Figure 3B) that was isolated in this study was found linked to greatest number of isolates. Other ST such as 744, 215 and 761 in this study were revealed as founder and were also found linked to other already reported isolates (Figure 3B, isolates are encircled). Surprisingly, geoBURST analysis revealed that most of the isolates of ST under study (ST88, ST744, ST215, ST761, ST1121, ST10005 and ST410 *etc*.) were all founders, while other such as ST2035, ST10002 and ST1080 *etc*. were demarcated as co-founders.

### Sequence compositional analysis

Sequence compositional analysis was performed in order to determine sequence diversity among the seven alleles used for MLST analysis. For this purpose, average GC contents, number of polymorphic sites, haplotype diversity, sites for synonymous and non-synonymous substitution and mean overall distance were determined and mentioned in Table 5. Selection analysis implemented in MEGA 7 programme indicated that all seven loci exhibited purifying selection, therefore, Z-test of selection was performed based on the purifying selection and the stat values are mentioned in Table 5. The mean GC contents of all seven loci were in the range of 50-55.9%, and number of polymorphic sites ranged from 9 to 113 (Table 5).

**Table 5:** Compositional characteristics of genes used in multilocus sequence typing of ESBL-producing *E. coli*

Locus	Length	G+C^a^	Poly. sites^b^	HD^c^	Ss^d^	Ns^e^	PS_prob_^f^	d/S.E^g^
*adk*	536	52.9	30	0.687	129.42	404.58	3.41/0.00	1.64/2.4
*fumc*	469	55.6	39	0.836	109.79	355.21	5.55/0.00	0.01/0.01
*gyrB*	460	52.8	113	0.886	108.58	350.42	4.96/0.00	0.02/0.01
*icd*	518	50.5	87	0.899	127.45	388.55	5.46/0.00	0.02/0.01
*mdh*	452	51.4	13	0.828	117.32	332.68	3.17/0.00	0.01/0.00
*purA*	478	52.7	9	0.818	123.70	350.30	2.71/0.00	0.01/0.00
*recA*	510	55.9	56s	0.708	124.33	382.67	4.56/0.00	0.01/0.00

^a^Average G+C content (%), ^b^No of polymorphic sites, ^c^HD = Haplotype diversity, ^d^Ss = Number of Synonymous sites, ^e^Number of Non synonymous sites, ^f^PS_prob_ = Stat value purifying selection and Probability, ^g^d/S.E = distance/standard error (d/S.E).

### Multi-locus sequence analysis (MLSA)

**Figure 4:**
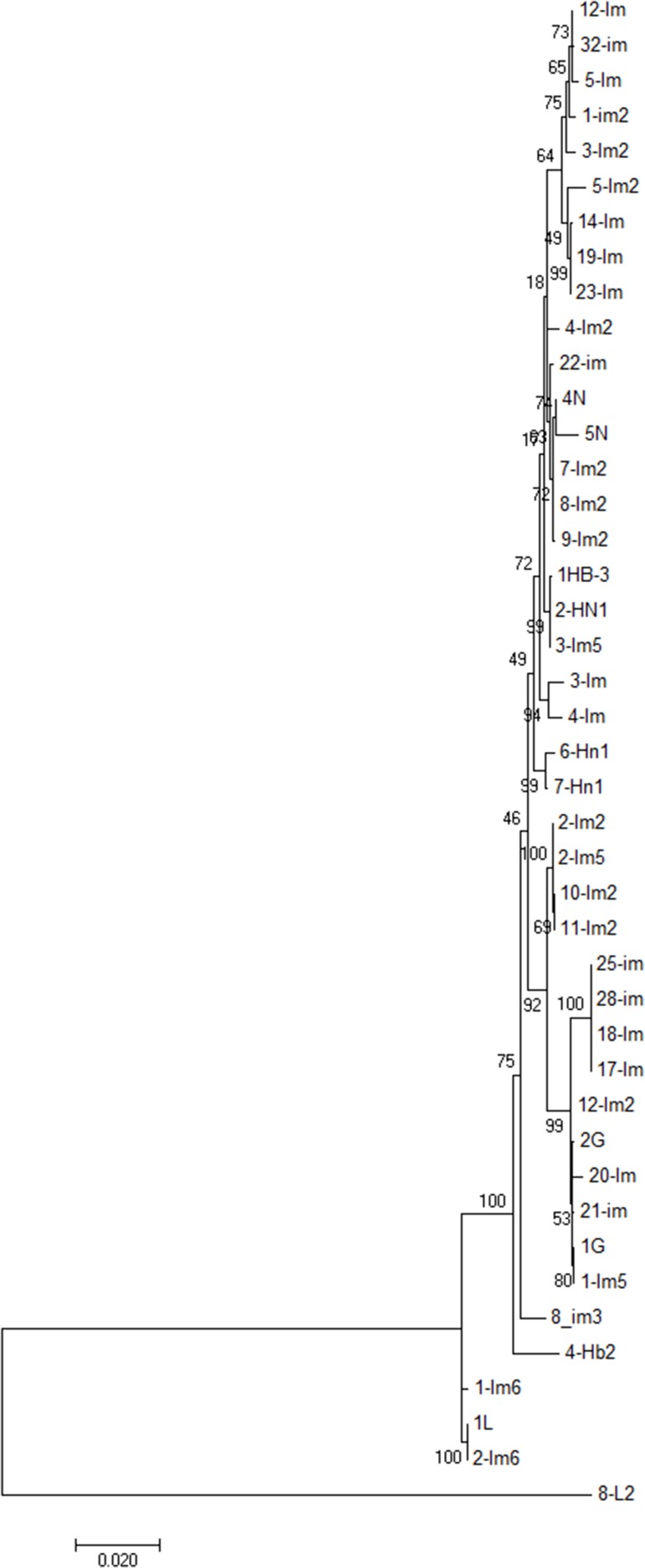
Phylogenetic analysis ESBL-producing *E. coli* isolates (n = 43) from bovine mastitis Phylogenetic tree was constructed from deduced concatenated sequences of the MLST allelic loci with maximum likelihood method using MEGA 7.0. Scale bar is indicated.

Although we characterized all isolates included in this study into phylogroups, this information does not provide crucial molecular features of the isolates relevant to epidemiological investigation, as well as not allowing accurate identification of species. Thus, to precisely identify species of the *E. coli* isolates under study we implemented MLSA, which is based on analysis of allelic nucleotide sequences used in MLST. Thus, by utilizing the concatenated nucleotide sequences obtained from typed strains (*n* = 43), phylogenetic analysis carried out by using multilocus sequence information of isolates. The maximum likelihood tree constructed using nucleotide sequences of seven loci revealed that study isolates were grouped into two clusters, but the larger cluster had diverse lineage (Figure 4) comprising multiple sub-clusters. A small cluster comprised only of 3 isolates, all originating from Inner Mongolia. The larger cluster was comprised of 42 (91%) isolates, however, unlike the small cluster, this was a tight cluster further comprised of small sub-clusters. An out-group isolate (8-L2) was clustered independently. Interestingly, isolates that could not be assigned with a ST due to a mismatch of at least one of the allelic number; and hence, were assigned with arbitrary numbers (10000-07; 3Im, 4-Im, 28-Im, 32-Im, 4N, 5N, 8L2 and 6-Hn1) (Table 4) were found distributed across all clusters. Furthermore, isolates were not clustered based on the origin or location. Two major sub-clusters, both comprised of a total of 14 isolates each, contained isolates from Guangdong, Hebei, Henan, and Inner Mongolia. However, isolates originating from Jiangsu and Inner Mongolia were clustered on distinct branches. Isolates from different regions were clustered together, which suggests that a connection between genetic background and sample origin could not be identified (Figure 4).

### Recombination analysis

**Figure 5:**
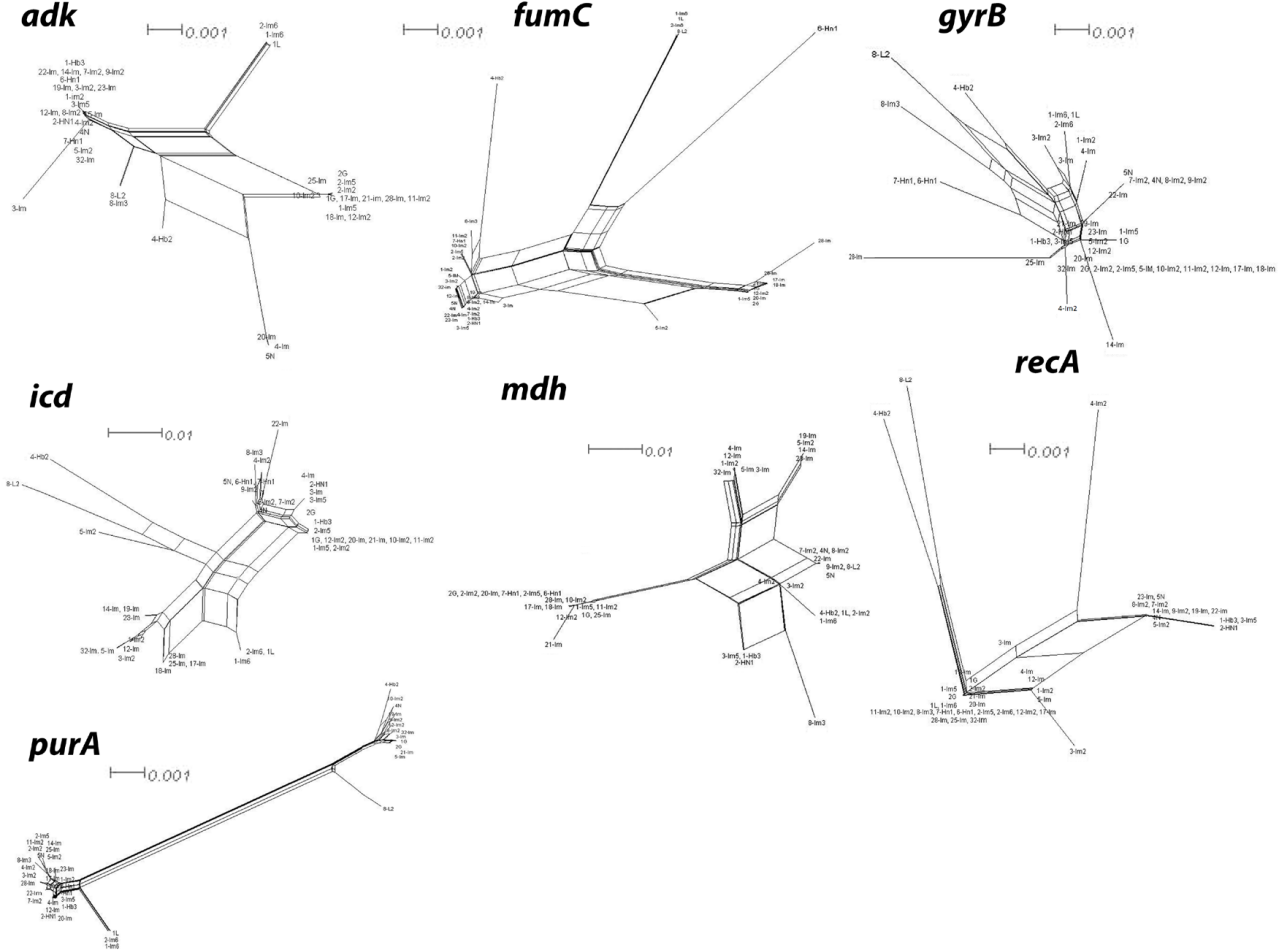
Split network analysis of seven individual multi-locus sequence typing (MLST) loci Split network analysis of seven individual MLST loci of *E. coli* isolates under study. Splits Tree4 has been used for creating network graphs by choosing neighbour-joining method. Multi-parallelogram formation with no tree like structures, as seen clearly in the case of *adk*, *fumC*, *icd, mdh* and *purA*, indicates that recombination has occurred. A tree like network suggests no recombination. Bootstrap values (not shown to avoid clutter) for majority of the parallelograms exceeding 80% indicating branching is significantly reliable between the strains (branch length of isolate 8-L2 is not drawn to scale). *E. coli* isolates IDs have been used as numbering in the figure indicate. For ST designations see Table 1. Bar scale is indicated (branch length of isolate 8-L2 is not drawn to scale).

**Figure 6:**
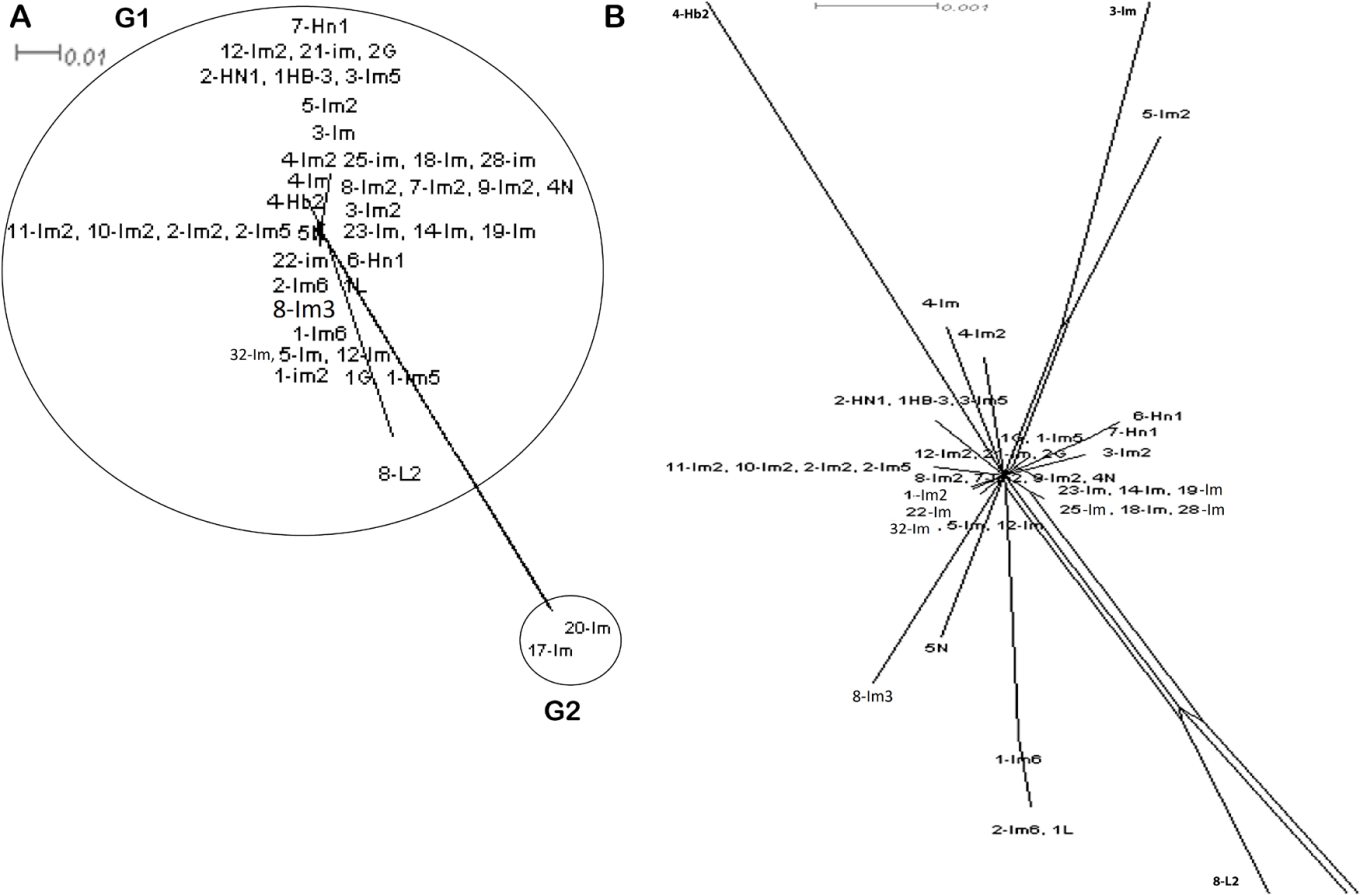
Split network analysis of all seven multi-locus sequence typing allelic loci concatenated sequences of 43 ST Split network analysis based on the concatenated sequence of MLST allelic locus of 43 ST of *E. coli* under study by adopting neighbour-net method implement in Split Tree. **(A)** Split network analysis of concatenated sequences of MLST allelic locus of 43 ST of *E. coli* isolates indicating a complex network-like structure suggesting several events of recombination. **(B)** Split network analysis of *E. coli* lineage belonging to Group 1 and primarily isolates of Inner Mongolia region of China province and reflecting multiparallelogram formation as well complex network-like indicating evidence of recombination. Some of the isolates like 5N, 2-Im6, 1L, 8-Im3 ad 5-Im-2 branched out of the main population network. Bootstrap values (not shown) for all branches are more than 80%. Bar scale is indicated above the figure (branch lengths of 8-L2, 3-Im and 4-Hb2 are not drawn to scale).

We performed split network analysis in order to investigate proof of recombination amongst the 43 isolates. Our analysis of 28 ST revealed various structures in the split graphs for the seven loci of MLST (Figure 5). The composition of split graphs of *adk*, *fumc*, *icd*, *gyrB* and *mdh* showed network-like parallelogram structures, indicating intergenic recombination happened during the evolutionary history of these genes. The fits were 95.3, 99.4, 97.7, 96.4, 99.2, 96.8 and 99.4 for *adk*, *fumc*, *gyrB, icd*, *mdh, purA* and *recA,* respectively, indicating the vast majority of information was consistent with the analysis. All of the parallelograms generated bootstrapping values greater than 50 with majority of them had bootstrapping value greater than 90, which suggests that the analysis were statistically robust. Surprisingly, none of the genes produced an explicit tree-like network which would have revealed these genes were of clonal descent and hence no recombination had occurred. Next, the split decomposition analysis of collective seven MLST loci revealed network-like structures with rays of different lengths (Figure 6). The fit was 78.9 (with LSFit=99.05), indicating that most information was in line with the analysis. The bootstrapping values for parallelogram generated were found greater than 80% suggesting branching is significantly reliable between the strains. All our tested isolates of 28 ST have been divided into two distinct groups (G1 and G2). Interestingly, both of these groups were completely disconnected from each other suggesting that the interspecies recombination had not happened. Nevertheless, G1, which was the largest group comprised of ST primarily from Inner Mongolia, displayed a parallelogram-shaped network revealing that interspecies intergenic recombination between the isolates of these groups had occurred during the course of evolution. ST3951 (isolate 8-L2) is distinct and disconnected from both groups, suggesting that recombination had not occurred between this isolate and the isolates of all other groups (Figure 6). Nevertheless, network structure of the G1 isolates further indicated rays of different lengths and a couple of disconnected isolates.

The *phi* test, a fast and statistically efficient method, is widely utilized for the analysis of recombination events. The resultant *P* value produced by applying *phi* test for all the 43 isolates of 28 ST is 0.0 (Mean: 0.191, Variance: 1.196, Observed: 0.065, *P*-value: 0.0) suggesting significant incidence of recombination across the whole population. Standard association index (*I_A_^S^*) calculates the homologous recombination by estimating the linkage disequilibrium among the seven MLST loci. When a population is at linkage equilibrium, the resultant *I_A_^S^* is expected to be zero. Analysis of our 43 *E. coli* isolates of 28 ST under this study yielded *I_A_^S^* of 0.2776 (*P* = < 0.001) (Supplementary Table 3). The *I_A_^S^* values are significantly different from zero, suggesting linkage disequilibrium among the alleles showing clonal relationship, and recombination was not adequate to break down the linkage disequilibrium.

## DISCUSSION

In this study, we highlight the features of 46 ESBL producers multi-drug resistant *E. coli* isolated from bovine mastitic milk samples from different provinces of China over the last two years (2015-2016). Among them, 39 isolates harboured CTX-M gene, while 22 and 8 isolates harboured TEM and SHV genes, respectively. These genes were associated with plasmid replicon types, primarily IncF. Interestingly, these resistant genes were transferable as suggested by our mating experiment. In addition, MLSA suggest two major lineages with a large lineage consisted of isolates primarily originated from Inner Mongolia. Split networking and genetic recombination analysis suggested intraspecies recombination events resulted into clonal diversity.

Antimicrobial resistance in Gram-negative bacteria is on the rise worldwide, particularly in *E. coli*, which constitute a majority of invasive Gram-negative isolates. More worrisome is the increasing reports of multi-drug resistant pathogenic *E. coli* from food producing animals that increases concerns for veterinary and public health [18]. Our study indicates an overall 25.41% of ESBL prevalence in *E. coli*, which is a greater prevalence of ESBL-producing *E. coli* from bovine mastitis as compared to previous reports from other countries [19, 20]. Possibly, the consistent selective pressure created by the long term use of antibacterial therapy during treatment of mastitis encouraged this rise of ESBL resistance. Our finding of higher prevalence (39/46) of *bla*_CTX-M_ genotypes are consistent with our previous report [4] and other reports from China and other parts of the world [3, 20]. Notably, the combination of *bla*_CTX-M_*+bla*_TEM_*+bla*_SHV_ has been rarely reported. We presume that this combination of resistance genes with other features associated with highly mobile plasmids types, such as IncF, insertion sequence elements or integrons are responsible for conferring MDR features and the rapid dissemination of these resistance determinants. Some of the isolates under study have been previously characterized for integrons and insertion sequence analysis indicated that integrons type 1 was the most prevalent type [4]. However, the ESBL gene was not found associated with integrons, rather it was found linked to IS*CR1* [4]. Similarly, all the new ESBL-producing isolates in the current study, as isolated from bovine mastitis, also belonged to class 1 integrons and harbored similar gene cassettes within the variable regions (data not shown). Thus, association of such mobile elements with the antibacterial-bacterial conferring resistant genes together with conjugative plasmids are extremely alarming. This study indicated that the commensal phylogenetic group A represented the major prevalent phylogroup harbouring ESBL genes and having multidrug resistance [21]. This is worrisome, as these commensals may serve as a reservoir for dissemination of resistance elements, and may in the future embrace virulent traits.

MLST is a precise molecular typing technique that has been successfully used to type more than seven thousand *E. coli* isolates available at MLST data base (http://mlst.warwick.ac.uk/mlst/dbs/Ecoli/). MLST enabled us to type and establish clonal relationship of our isolates and multilocus sequence analysis facilitated us to infer phylogenetic relationship amongst the isolates and species of *E. coli* isolates. MLST analysis of the 43 successfully typeable *E. coli* strains revealed the extent of diversity within *E. coli* population from the world's most populous country, with a high density of human and animal populations, further coupled with the extensive application of antimicrobial therapy in food animals [4]. Our analysis indicated that all 43 isolates are distributed in 28 ST, of which 9 could not be assigned as their allelic profile did not match with the available database. Interestingly, all 9 unassigned isolates reflected a single allele mismatch with the database. These isolates might be of novel types, and hence further studies are under way for whole genome sequencing to identify the ST of these isolates. MLSA analysis indicated that all ST were clustered into two lineages, however, both lineages (a smaller tight lineage and a larger diverse lineage) consisted of isolates from diverse origins and various locations. Therefore, we assumed that the current MLST data of *E. coli* isolates was descriptive at the strain level, however might not be used to infer a relationship between the genetic backgrounds and origin of the isolates. Interestingly, few isolates from Inner Mongolia were included in the smaller tight lineage which consisted of isolates from almost all other regions we sampled; yet, isolates from Inner Mongolia, Hebei, Liaoning and Henan were also clustered into the bigger diverse group suggesting that these ESBL producers can be more diverse or ancestral. Furthermore, three identical ST, ST410, from same farm of Inner Mongolia isolated at intervals suggesting chronic infection, which is a classic pattern of mastitis.

Notably, ST410 represents 10% (4/43) of the typeable population reported in this study. All these isolates originated from Inner Mongolia, the province with very dense farming, however, surprisingly, one of the isolates belong to phylogroup D and B1 each, while two isolates belonged to phylogroup A, reflecting diversity. Further, these isolates harbouring IncFIB (*n* = 4), IncFrep (*n* = 4), IncK/B (*n* = 1) and IncN (*n* = 2) plasmid replicon types, respectively. Additionally these isolates were carrying diverse types of ESBL genes (CTX-M-55+ TEM-1(*n* = 2), CTX-M-15+ SHV-1 (*n* = 1), CTX-M-14 (*n* = 1), while they were all resistant to most commonly used β-lactam and non-β-lactam drugs (Table 1). Of note, ST410 is distributed worldwide and is reported in Spain to carry CTX-M and virulence genes isolated from meat and clinical samples [22], in Greece harbouring CTX-M-1 and FIIK replicon type [23], in Pakistan and England producing NDM-1 carbapenemase [24] and China [25]. Other ST that were very prevalent included ST361, ST4085, ST117 and ST58. Recently, ST10, ST58, ST69 *E. coli* producing CTX-15 type of ESBL were also reported in Lebanese cattle [26]. The geoBURST analysis indicated that of the prevalent ST, primarily ST410 and ST361 including the ST88 (less prevalent) were demarcated as founders. These ST have been isolated from different areas, but primarily from Inner Mongolia. ST88 has been reported worldwide, primarily in Europe [27] and Asia including China [28], making it a well-known species. Of note, none of our *E. coli* strains belonged to ST131, which is the most prevalent ST in human beings. Nevertheless, *E. coli* producing ESBL CTX-M-15 belonging to non-ST131 are also reported in human beings such as ST410, ST38 and ST10 indicating worldwide presence [29, 30]. This suggests that these globally disseminated clones and isolates from our study are closely related genetically. Finally, analysis of genetic recombination suggests that despite few allelic loci exhibiting intergenic recombination, genotyping diversity generated is clonal in nature and to investigate interspecies recombination events a larger collection of strains is required as with the current low number of isolates no strong evidence of interspecies recombination was observed.

## MATERIALS AND METHODS

### Statement of ethics

Prior approval to carry out the current work was obtained (dated 26/10/2015) from the departmental ethical committee at College of Veterinary Medicine, China Agricultural University, Beijing (CAU), and it was carried out according to the standard ethical guide lines implemented at CAU. Additionally, milk samples from the mastitic cows were collected according to the standard procedures of National Mastitis Council (NMC) and with proper consultation of the dairy farm's owner or administration.

### Milk sample collection

Milk samples were collected, as per NMC guide lines, from lactating cows (one sample per cow) suffering from bovine mastitis from 69 dairy herds in 16 provinces of China over a period of two years (2015-2016). These samples were taken in sterile bottles (50 mL) and transported to our mastitis reference laboratory in ice boxes for the identification of causative agents of mastitis and antibiotic susceptibility testing as described previously [4]. Clinical mastitis was diagnosed on the basis of visual abnormality/inflammation of the mammary gland or its milk secretion, and subclinical mastitis was detected by measuring somatic cell count [32]. The details of collected samples are present in the Supplementary Table 1.

### Isolation and identification of isolates and ESBL phenotypes

A 10 μL of milk sample was spread by a sterile cotton swab on the tryptose soy agar (TSA; Difco™, Becton Dickison, Sparks, MD) augmented with defibrinated sheep blood (5%) and incubated for 24 h at 37 °C. The Gram negative, as revealed from standard gram staining, were further streaked onto the MacConkey agar (Difco™) and Eosin methylene blue agars (Difco™) and incubated for further 18-24 h. The dark pink colonies on MCA and green metallic sheen colonies on Eosin methylene blue were the presumptive *E. coli*, which were further confirmed by API-20E kit (bioMérieux, Marcy I’Etoile, France) and PCR assay. Our previous protocol for the isolation and confirmation of ESBL-producing *E. coli* was adopted for the detection of ESBL-phenotypes [31]. Briefly, the ESBL-producing isolates were confirmed by double-disc synergy test [32], after the initial growth of *E. coli* on MacConkey agar supplemented with cefotaxime (2 mg/L). All verified isolates were maintained at -80 °C in brain heart infusion broth (Sigma-Aldrich) containing 30% glycerol until further processing.

### *In vitro* antibiotic susceptibility testing

Antibiotic susceptibility profiles of all the clinical isolates were tested against a panel of eighteen different antibiotics using the Kirby-Bauer disk diffusion method on Muller-Hinton agar (Difco™) as per standard guidelines of CLSI [32]. The antimicrobial agents included ampicillin, amoxicillin/clavulanic acid, cephalexin, cefaclor, cefoxatin, cefotaxime, ceftazidime, cefepime, aztreonam, meropenem, nalidixic acid, chloramphenicol, ciprofloxacin, trimethoprim/sulphamethoxazole, tetracycline, gentamicin, cefotaxime/ clavulanic acid and ceftazidime/clavulanic acid (BD BBL™, Oxoid™). Minimum inhibitory concentrations by broth microdilution method of the selected common antibiotics (ampicillin, cefazolin, cefoxatin, cefotaxime, ceftriaxone, ciprofloxacin, norfloxacin, trimethoprim (BBI Life Sciences Corporation, Shanghai, China), amikacin, chloramphenicol, gentamicin, kanamycin and tetracycline (Amresco, OH, USA)) for the ESBL-producing *E. coli* were also determined in accordance with the guidelines of the CLSI [32]. ESBL negative quality control strain was *E. coli* ATCC25922, whereas, *Klebsiella pneumoniae* ATCC700603 was used as ESBL positive control strain [32]. Multidrug resistant isolates were those found resistant to at least three or more categories of antimicrobial agents.

### Molecular characterization of ESBL-producing isolates

The genomic and plasmid DNA from the ESBL-producing isolates were extracted by using a commercial kit (QIAamp DNA minikit, Qiagen, Germany). The ESBL encoding genes were detected using polymerase chain reaction (PCR) assay, which included *bla*_CTX-M_, *bla*_SHV_, *bla*_TEM_ [33]. The primers used in this study are listed in Supplementary Table 2. The reaction mixture (50 μL) consisted of 5 μL of 10X*TransStart* ^®^ *Top Taq* buffer, 4 μL of 2.5mM deoxynucleoside triphosphate (dNTPs; Transgen, Beijing, China), 0.5 μL of *TransStart* ^®^ *Top Taq* DNA polymerase (2U; Transgen, Beijing, China), 2 μL of template DNA, 1 μL of each primer (10 μM; Sunbiotech, Beijing) and 36.5 μL of ultra-pure distilled water. The amplified PCR product was purified (TIANquick Midi purification Kit) and bi-directionally sequenced by the primers used for amplification using an automated sequencer (ABI 3730; Applied Biosystems, Foster City, CA, USA). The obtained sequences were submitted to NCBI data base for homology study (http://www.ncbi.nlm.nih.gov/BLAST/). The already confirmed strains [4] and *Klebsiella pneumoniae* ATCC 700603 were used as positive control strains in PCR assays. Additionally, the phylogenetic groups of all isolates were detected using the triplex PCR scheme of Clermont et al. [31].

### Plasmid replicon typing

PCR-based plasmid replicon typing, using five multiplex and three singleplex PCR assays, was carried out as described previously by Carattoli et al. [34]. Primer sequences, their amplicon sizes and annealing temperatures are mentioned in Supplementary Table 2.

### Resistance transfer experiment

To investigate the transferability of the ESBL encoding genes and to know that these genes were located on plasmids, a conjugation assay was carried out for randomly selected twelve ESBL-producing *E. coli* (donor strains) using the sodium azide-resistant *E. coli* J53 strain as recipients. Mating assay was performed on Muller-Hinton agar (Oxoid) plates as described previously [35]. The transconjugates were finally obtained on Muller-Hinton agar supplemented with cefotaxime (2 mg/L) and sodium azide (150 mg/L). All transconjugates were screened for antimicrobial susceptibility by disc diffusion method and MIC of cefotaxime and ceftazidime, ESBL phenotypes and genotypes detection and plasmid replicon typing by methods described above.

### Multilocus sequence typing of ESBL-producing *E. coli*

To determine the evolutionary relationships of the ESBL-producing isolates, MLST of all the isolates was performed by PCR amplification of the seven housekeeping genes (*adk, fumC, gyrB, icd, mdh, purA* and *recA*) following guidelines of the MLST databases (http://mlst.warwick.ac.uk/mlst/dbs/Ecoli/) as proposed by Wirth et al. (2006) [15]. Allelic profile was determined by the specific allelic profiles (combination of alleles) and ST were assigned based on the combination of seven alleles at MLST database. Sequence types were assigned an arbitrary number if their allelic profile did not match with the available database.

### Population structure analysis

To determine, the clonal complexes of ST eBURST algorithm (htpp://ebusrst.mlst.net) was used by applying eBURSTv3, while Burst group/Clonal complexes were defined as the ST sharing six or more common loci. Furthermore, double locus variants and singletons (ST with at least two allelic mismatches with all other ST) were also defined by eBURST analysis [36]. The eBURST algorithm is structured on a model based on bacterial evolution. In such a model, a single ancestor founder ST gives rise to a subset of closely related ST by a series of continuous diversification through the process of evolution. The relationship between the ST generated in this report with the available ST in the global MLST data base (https://pubmlst.org/bigsdb?db=pubmlst_mlst_seqdef&page=query) was assessed by using geoBURST [37]. Sequence alignments of all seven housekeeping genes of concatenated sequences of all tested isolates under study was performed using MUSCL implemented in MEGA 7 and maximum likelihood tree was developed by choosing General Time Reversible model, Gamma distributed and Invariant sites (G+1) with 1000 bootstrap replication implemented in MEGA version 7.0 [38].

### Sequence compositional analysis

The number of polymorphic sites, haplotype diversity, synonymous and non-synonymous sites were calculated by using DnaSP version 5.10 [39]. Average GC contents of each of the seven alleles, mean overall distance and status of purifying selection were calculated using MEGA version 7 after correcting the frame of the open reading frames of housekeeping genes, when required.

### Split network and recombination analysis

The graphs and analysis of split network of all ST as well as individual locus were produced by applying neighbour-net method using SplitsTree4 [40]. The statistical significance for the parameters to perform evolutionary analysis by SplitsTree has been tested using bootstrap resampling at 1000 and level of fits. The pair wise homoplasy index (*phi*) test [41] integrated in SplitsTree4 [40] for recombination was performed, whereby the *P* value < 0.05 concluding recombination event has occurred.

Linkage disequilibrium analysis based on the allelic profile data obtained for MLST was tested by calculating the standardized index of association (*I_A_^S^*) using LIAN v3.75 [42] at http://guanine.evolbio.mpg.de/cgi-bin/lian/lian.cgi.pl. The Monte Carlo method was used in order to evaluate the null hypothesis of complete linkage equilibrium (*I_A_^S^*>0; presence of linkage disequilibrium or clonality) by choosing 10,000 iterations on the allelic profile of under study isolates.

## CONCLUSIONS

Altogether, resistance profiles, analysis of MIC, plasmid replicon typing, MLST and MLSA analysis provided a great insight into the nature of resistance profiles, mode of dissemination and diversity amongst the *E. coli* ESBL-producers isolated from bovine mastitis samples in China. Our isolates, particularly those unassigned ST, are quite interesting and whole genome sequencing of few candidate isolates will certainly help to understand resistance, virulence associated loci and genomic diversity variation by horizontal gene transfer. Moreover, the current study should be expanded to a larger sample size including other food animal species and community hospitals for effective epidemiological and surveillance reports.

## SUPPLEMENTARY MATERIALS TABLES




