# Supplementary material for: Characteristics and genetic diversity of multi-drug resistant extended-spectrum beta-lactamase (ESBL)-producing Escherichia coli isolated from bovine mastitis
Source: Oncotarget. 2017 Oct 4;8(52):90144–63. doi: 10.18632/oncotarget.21496 (PMC5685738; doi:10.18632/oncotarget.21496)
Supplement: Supplementary file 1 [file oncotarget-08-90144-s001.pdf]

Characteristics and genetic diversity of multi-drug resistant extended-spectrum beta-lactamase (ESBL)-producing *Escherichia coli* isolated from bovine mastitis

SUPPLEMENTARY MATERIALS

Supplementary Table 1: Details of milk samples collection from mastitic cows located various in various provinces and isolation of extended-spectrum beta-lactamase-producing *E. coli*

Locations	Dairy Herds sampled	Samples collected	*E. coli* isolated	ESBL-producers
Anhui	2	78	7	0
Beijing	5	72	10	0
Fujian	1	12	0	0
Guangdong	3	124	7	2
Hebei	14	220	34	2
Heilongjiang	4	73	7	1
Henan	5	43	5	4
Inner-Mongolia	14	446	52	33
Jiangsu	2	27	7	2
Liaoning	3	63	10	1
Ningxia	4	97	19	1
Shaanxi	1	13	2	0
Shandong	4	83	8	0
Shanxi	1	6	0	0
Shanghai	4	59	10	0
Tianjin	2	24	3	0
Total	69	1440	181 (12.57%)	46 (25.41%)

Supplementary Table 2: List of primers used in this study

See Supplementary File 1

Supplementary Table 3: eBurst analysis of the 43 *E. coli* isolates

No. isolates = 43 | No. STs = 28 | No. re-samplings for bootstrapping = 1000

No. loci per isolate = 7 | No. identical loci for group def = 6 | No. groups = 5

Group 1: No. Isolates = 6 | No. STs = 3 | Predicted Founder = 88

ST	FREQ	SLV	DLV	TLV	SAT	Average Distance	ST Bootstrap Group	Subgrp
88	1	2	0	0	0	1.0	32%	0%
410	4	1	1	0	0	1.5	0%	0%
10005	1	1	1	0	0	1.5	0%	0%

Group 2: No. Isolates = 3 | No. STs = 2 | Predicted Founder = None

ST	FREQ	SLV	DLV	TLV	SAT	Distance
744	2	1	0	0	0	1.0
761	1	1	0	0	0	1.0

Group 3: No. Isolates = 2 | No. STs = 2 | Predicted Founder = None

ST	FREQ	SLV	DLV	TLV	SAT	Distance
10007	1	1	0	0	0	1.0
540	1	1	0	0	0	1.0

Group 4: No. Isolates = 3 | No. STs = 2 | Predicted Founder = None

ST	FREQ	SLV	DLV	TLV	SAT	Distance
2008	2	1	0	0	0	1.0
10004	1	1	0	0	0	1.0

Group 5: No. Isolates = 3 | No. STs = 2 | Predicted Founder = None

ST	FREQ	SLV	DLV	TLV	SAT	Distance
1121	2	1	0	0	0	1.0
10002	1	1	0	0	0	1.0

Singletons: size 17

10006; 69; 10003; 215; 2035; 10001; 4085; 10000; 5442; 361; 392; 117; 2521; 58; 5746; 1080; 3476
