# Supplementary material for: Characteristics and genetic diversity of multi-drug resistant extended-spectrum beta-lactamase (ESBL)-producing Escherichia coli isolated from bovine mastitis
Source: Oncotarget. 2017 Oct 4;8(52):90144–63. doi: 10.18632/oncotarget.21496 (PMC5685738; doi:10.18632/oncotarget.21496)
Supplement: Supplementary file 2 [file oncotarget-08-90144-s002.docx]

**Table S2:** List of primers used in this study.

| **Primers** | **Sequence (5’ to 3’)** | **Target sites/g-enes** | **Ann-ealing tempe-rature** | **Amp-licons size** | **References** |
| --- | --- | --- | --- | --- | --- |
| ***ESBL genes*** |  |  |  |  |  |
| CTX-MA  CTX-MB | CGC TTT GCG ATG TGC AG  ACC GCG ATA TCG TTG GT | *bla*_CTX-M_ | 54 ^o^C | 550-bp | ([Villegas et al., 2004](#_ENREF_40)) |
| SHV-F  SHV-R | GGG TTA TTC TTA TTT GTC GC  TTA GCG TTG CCA GTG CTC | *bla*_SHV_ | 58 ^o^C | 567-bp | ([Chang et al., 2001](#_ENREF_6)) |
| TEM-F  TEM-R | ATA AAA TTC TTG AAG ACG AAA  GAC AGT TAC CAA TGC TTA ATC | *bla_TEM_* | 56 ^o^C | 1086-bp | ([Yao et al., 2007](#_ENREF_43)) |
| ***E. coli*-specific** |  |  |  |  |  |
| UAL  UAR | TGG TAA TTA CCG ACG AAA ACG GC  ACG CGT GGT TAC AGT CTT GCG | *uidA* | 62 ^o^C | 147-bp | ([Tantawiwat et al., 2005](#_ENREF_37)) |
| ***Phylo-groups*** |  |  |  |  |  |
| ChuA-F  ChuA-R | GAC GAA CCA ACG GTC AGG AT  TGC CGC CAG TAC CAA AGA CA | ChuA | 55 ^o^C | 279-bp | ([Clermont et al., 2000](#_ENREF_9)) |
| YjaA-F  YjaA-R | TGA AGT GTC AGG AGA CGC TG  ATG GAG AAT GCG TTC CTC AAC | YjaA | 55 ^o^C | 211-bp | ([Clermont et al., 2000](#_ENREF_9)) |
| TspE4C2-F  TspE4C2-R | GAG TAA TGT CGG GGC ATT CA  CGC GCC AAC AAA GTA TTA CG | *TspE4C2* | 55 ^o^C | 152-bp | ([Clermont et al., 2000](#_ENREF_9)) |
| ***PBRT* primers*** |  |  |  |  |  |
| HI1-F  HI1-R | GGAGCGATGGATTACTTCAGTAC  TGCCGTTTCACCTCGTGAGTA | parA-parB | 58 ^o^C | 471-bp | (Carattoli et al., 2005) |
| HI2 -F  HI2- R | GGCTCACTACCGTTGTCATCCT  CGAAAGCCGGACGGCAGAA | RNAI | 58 ^o^C | 644-bp | (Carattoli et al., 2005) |
| I1-F  I1-F | CGAAAGCCGGACGGCAGAA  TCGTCGTTCCGCCAAGTTCGT | iterons | 58 ^o^C | 139-bp | (Carattoli et al., 2005) |
| X -F  X -R | AACCTTAGAGGCTATTTAAGTTGCTGAT  TGAGAGTCAATTTTTATCTCATGTTTTAGC | ori ꭚ | 58 ^o^C | 376-bp | (Carattoli et al., 2005) |
| L/M-F  L/M-F | GGATGAAAACTATCAGCATCTGAAG  CTGCAGGGGCGATTCTTTAGG | repA, B, C | 58 ^o^C | 785-bp | (Carattoli et al., 2005) |
| N-F  N-R | GTCTAACGAGCTTACCGAAG  GTTTCAACTCTGCCAAGTTC | repA | 58 ^o^C | 559-bp | (Carattoli et al., 2005) |
| FIA- F  FIA- R | CCATGCTGGTTCTAGAGAAGGTG  GTATATCCTTACTGGCTTCCGCAG | iterons | 58 ^o^C | 462-bp | (Carattoli et al., 2005) |
| FIB –F  FIB -R | GGAGTTCTGACACACGATTTTCTG  CTCCCGTCGCTTCAGGGCATT | repA | 58 ^o^C | 702-bp | (Carattoli et al., 2005) |
| W-F  W-R | CCTAAGAACAACAAAGCCCCCG  GGTGCGCGGCATAGAACCGT | repA | 58 ^o^C | 242-bp | (Carattoli et al., 2005) |
| Y-F  Y-R | AATTCAAACAACACTGTGCAGCCTG  GCGAGAATGGACGATTACAAAACTTT | repA | 58 ^o^C | 765-bp | (Carattoli et al., 2005) |
| P-F  P-R | CTATGGCCCTGCAAACGCGCCAGAAA  TCACGCGCCAGGGCGCAGCC | iterons | 58 ^o^C | 534-bp | (Carattoli et al., 2005) |
| FIC –F  FIC -R | GTGAACTGGCAGATGAGGAAGG  TTCTCCTCGTCGCCAAACTAGAT | repA2 | 58 ^o^C | 262-bp | (Carattoli et al., 2005) |
| A/C -F  A/C -R | GAGAACCAAAGACAAAGACCTGGA  ACGACAAACCTGAATTGCCTCCTT | repA | 58 ^o^C | 465-bp | (Carattoli et al., 2005) |
| T-F  T-R | TTGGCCTGTTTGTGCCTAAACCAT  CGTTGATTACACTTAGCTTTGGAC | repA | 58 ^o^C | 750-bp | (Carattoli et al., 2005) |
| FIIS –F  FIIS -R | CTGTCGTAAGCTGATGGC  CTCTGCCACAAACTTCAGC | repA | 58 ^o^C | 270-bp | (Carattoli et al., 2005) |
| FrepB-F  FrepB-R | TGATCGTTTAAGGAATTTTG  GAAGATCAGTCACACCATCC | RNAI/repA | 58 ^o^C | 270-bp | (Carattoli et al., 2005) |
| K/B -F  K/B-R | GCGGTCCGGAAAGCCAGAAAAC  TCTTTCACGAGCCCGCCAAA | RNAI | 58 ^o^C | 160 bp | (Carattoli et al., 2005) |
| B/O-F  B/O-R | GCGGTCCGGAAAGCCAGAAAAC  TCTGCGTTCCGCCAAGTTCGA | RNAI | 58 ^o^C | 159 bp | (Carattoli et al., 2005) |
| ***MLST primers*** |  |  |  |  |  |
| Adk-F  Adk-R  Adk-F1  Adk-R1 | ATTCTGCTTGGCGCTCCGGG  CCGTCAACTTTCGCGTATTT  TCATCATCTGCACTTTCCGC  CCAGATCAGCGCGAACTTCA | *adk* | 54° C | 583 bp | (Wirth et al., 2006) |
| FumC-R1  FumC-F  FumC-R | TCCCGGCAGATAAGCTGTGG  TCACAGGTCGCCAGCGCTTC  GTACGCAGCGAAAAAGATTC | *fumC* | 54° C | 806 bp | Wirth et al., 2006) |
| GyrB-F  GyrB-R1  GyrB-R | TCGGCGACACGGATGACGGC  GTCCATGTAGGCGTTCAGGG  ATCAGGCCTTCACGCGCATC | *gyrB* | 60 ° C | 911 bp | Wirth et al., 2006) |
| Icd-F  Icd-R | ATGGAAAGTAAAGTAGTTGTTCCGGCACA  GGACGCAGCAGGATCTGTT | *icd* | 54° C | 878 bp | Wirth et al., 2006) |
| *Mdh-F*  *Mdh-R*  *Mdh-F1*  *mdh-R1* | ATGAAAGTCGCAGTCCTCGGCGCTGCTGGCGG  TTAACGAACTCCTGCCCCAGAGCGATATCTTTCTT  AGCGCGTTCTGTTCAAATGC  CAGGTTCAGAACTCTCTCTGT | *mdh* | 60 °C | 932 bp | Wirth et al., 2006) |
| *purA-F1*  *purA-F*  *purA-R* | TCGGTAACGGTGTTGTGCTG  CGCGCTGATGAAAGAGATGA  CATACGGTAAGCCACGCAGA | *purA* | 54° C | 816 bp | Wirth et al., 2006) |
| *recA-R1*  *recA-F*  *recA-F1*  *recA-R* | AGCGTGAAGGTAAAACCTGTG  CGCATTCGCTTTACCCTGACC  ACCTTTGTAGCTGTACCACG  TCGTCGAAATCTACGGACCGGA | *recA* | 58° C | 780 bp | Wirth et al., 2006) |

F, forward; R, reverse; PBRT, PCR-based plasmid replicon typing
